# Autophagy-Related Proteins (ATGs) Are Differentially Required for Development and Virulence of *Sclerotinia sclerotiorum*

**DOI:** 10.3390/jof11050391

**Published:** 2025-05-19

**Authors:** Thilini Weerasinghe, Josh Li, Xuanye Chen, Jiayang Gao, Lei Tian, Yan Xu, Yihan Gong, Weijie Huang, Yuelin Zhang, Liwen Jiang, Xin Li

**Affiliations:** 1 Michael Smith Laboratories, University of British Columbia, Vancouver, BC V6T 1Z4, Canada; tw1994@student.ubc.ca (T.W.); joshli@student.ubc.ca (J.L.); lois.chen@botany.ubc.ca (X.C.); lei.tian@uni-goettingen.de (L.T.); yihan.gong@mail.utoronto.ca (Y.G.); weijie.huang@msl.ubc.ca (W.H.); 2 Department of Botany, University of British Columbia, Vancouver, BC V6T 1Z4, Canada; 3 School of Life Sciences, Chinese University of Hong Kong, Hong Kong, China; gaojybio@gmail.com (J.G.); ljiang@cuhk.edu.hk (L.J.); 4 The College of Life Sciences, Sichuan University, Chengdu 610064, China; xuyan0614@163.com (Y.X.); yuelin.zhang@scu.edu.cn (Y.Z.)

**Keywords:** fungal pathogens, *Sclerotinia sclerotiorum*, ATG, autophagy, Pexophage, futant screen, next-generation sequencing, forward genetic analysis, UV mutagenesis

## Abstract

*Sclerotinia sclerotiorum* is a devastating fungal pathogen that can colonize numerous crops. Despite its economic importance, the regulation of its development and pathogenicity remains poorly understood. From a forward genetic screen in *S. sclerotiorum*, six UV mutants were identified with loss-of-function mutations in *SsATG1*, *SsATG2*, *SsATG4*, *SsATG5*, *SsATG9*, and *SsATG26*. Functional validation through gene knockouts revealed that each *ATG* is essential for sclerotia formation, although the morphology of appressoria was not significantly altered in the mutants. Different levels of virulence attenuation were observed among these mutants. Autophagy, monitored using GFP-ATG8, showed dynamic activities during sclerotia development. These findings suggest that macroautophagy and pexophagy contribute to sclerotia maturation and virulence processes. Future work will reveal how autophagy controls target organelle or protein turnover to regulate these processes.

## 1. Introduction

Microbial diseases caused by fungi pose a significant threat to global food security, causing yield and quality losses of crops. The ascomycete *Sclerotinia sclerotiorum* (Lib.) de Bary infects a wide range of host plants, including many that are economically important [1]. It is one of the most devastating soilborne pathogens for canola, leading to approximately $200 million losses in the United States and 10–20% yield losses in China [2,3]. Additionally, *S. sclerotiorum* can infect crops including sunflower, soybean, tomato, lettuce, and onion [2]. Its life cycle features the formation of a resting structure termed sclerotium, which can germinate myceliogenically to infect nearby tissues or carpogenically to release airborne ascospores capable of infection [3,4]. In both scenarios, fungal hyphae differentiate into appressoria to penetrate the plant host surface [5]. Understanding the genetic mechanisms for how this fungus develops and proliferates in host plants will be essential for the development of sustainable strategies for crop protection.

Autophagy is a lysosome-/vacuole-mediated cellular degradation process in eukaryotes. It is highly conserved and can be subdivided into macroautophagy and microautophagy [6]. Macroautophagy involves enclosing large cytoplasmic portions within double-membrane vesicles called autophagosomes [7], whereas microautophagy captures smaller cytoplasmic contents directly through lysosomal membrane invagination [8,9]. Both pathways generally function as non-selective responses to nutrient deprivation, engulfing random portions of the cytoplasm. While under nutrient-rich conditions, selective autophagy can occur. For instance, the cytoplasm to vacuole targeting (Cvt) and pexophagy pathways can specifically target hydrolases and peroxiosomes, respectively [10,11], eliminating unneeded substances or removing harmful agents, such as reactive oxygen species [12,13].

For yeast macroautophagy, target of rapamycin complex 1 (TORC1) acts as a kinase to phosphorylate ATG13. Under starvation, TORC1 is inactivated, leading to the dephosphorylation of ATG13 [14]. Dephosphorylated ATG13 then binds directly to the ATG1 protein kinase and associates with the ATG17-ATG31-ATG29 complex via ATG17 to form the ATG1 kinase complex [15,16,17]. Subsequently, ATG9 is phosphorylated by ATG1 and recruited to the Phagophore Assembly Site (PAS) by ATG13 and ATG17, delivering the phospholipids necessary for initiating phagophore formation [18,19,20]. The newly formed phagophore expands in size and engulfs cargoes, ultimately forming sealed double-membrane autophagosomes. ATG18 is a phosphoinositide-binding protein that can bind phosphatidylinositol3-phosphate (PI3P) to target PAS and form an ATG18 complex together with ATG2 [21,22,23]. The ATG18 complex tethers phagophore membranes to the endoplasmic reticulum (ER) for autophagosome formation [24]. ATG12 is a ubiquitin-like protein that becomes covalently conjugated to ATG5 by ATG7 (E1-like activating enzyme) and ATG10 (E2-like conjugating enzyme) [14]. Then ATG12-ATG5 forms an ATG12-ATG5-ATG16 complex, which exhibits E3-like enzyme activity and recruits the ATG8 conjugation system to the PAS [14,25,26]. ATG8 is a ubiquitin-like protein cleaved by the protease ATG4, exposing a C-terminal glycine residue of ATG8 [27]. This glycine residue is then conjugated to phosphatidylethanolamine (PE), a lipid component of membranes, through a ubiquitin-like conjugation system involving the ATG7 and the E2-like enzyme ATG3 [28]. ATG8–PE facilitates the expansion of the phagophore and is involved in the closure of the autophagosomal membrane [29].

In filamentous fungi, autophagy is not only required for cell component degradation and recycling but also plays critical roles in development and pathogenesis. For example, in the rice blast fungus *Magnaporthe oryzae*, MoATG3, MoATG9, and MoATG14 contribute to the buildup of internal turgor pressure in the appressorium necessary for host penetration [30,31]. In *S. sclerotiorum*, several autophagy-related genes, including *SsATG1*, *SsATG15*, and *SsATG22*, are upregulated when grown in fermentation broth containing the biocontrol agent *Bacillus amyloliquefaciens* [32]. Additionally, deletion of *SsATG8* or its selective autophagy cargo receptor *SsNBR1* resulted in no sclerotia formation, reduced pathogenicity and significantly reduced oxalic acid (OA) levels [33]. Despite these findings, the precise mechanisms by which autophagy contributes to developmental processes in *S. sclerotiorum* remain unclear.

In this study, we aimed to identify mutants with defects in sclerotia formation through ultraviolet (UV) mutagenesis-based forward genetic screens [4]. Six UV-induced mutants are reported, and their causal genes were identified by next-generation sequencing (NGS). *SsATG1*, *SsATG9*, *SsATG2*, *SsATG5*, *SsATG4*, and *SsATG26* were considered to be the causal gene of UV mutants, which were later confirmed by non-sclerotia-forming phenotypes in the corresponding deletion knockout (KO) mutants. Changes in virulence were observed to different levels in some of the KO mutants. These results reveal the distinct roles of these *ATG*s during development and infection processes in *S. sclerotiorum*.

## 2. Materials and Methods

### 2.1. Fungal Strains of S. sclerotiorum and Culture Conditions

The wild-type strain *S. sclerotiorum* 1980 (WT) was generously provided by Dr. J. Rollins from the University of Florida (Gainesville, USA). All mutant strains derived from it were grown on potato dextrose agar (PDA, Shanghai Bio-Way Technology, Shanghai, China) at room temperature. The mutant strains include the oxalic acid biosynthesis deficient mutant *Ssoah1* with mutation in the *OAH1* gene [34,35] and the mutants generated from UV mutagenesis: M133, R240, P20B12, P18D4, LL247, and P38E2. The mutated genes in the UV mutants are included in Table 1. The mutants were stored on PDA slants at 4 °C for up to 6–12 months or preserved in 15% glycerol in a −80 °C freezer for long-term storage. *S. sclerotiorum* KO mutants were selected and purified on PDA with hygromycin B (Sigma, Carlsbad, CA, USA) at a final concentration of 50 µg/mL.

### 2.2. UV Mutagenesis

The UV mutagenesis screening protocol was adapted from one previously reported [4]. In short, ascospores collected from carpogenically germinated sclerotia [36] were resuspended in PDB at 10^4^ spores per milliliter and 300 µL of suspension was spread on PDA plates (90 mm diameter). These plates were subjected to UV radiation (TL-2000 Ultraviolet Translinker, Analytik Jena AG, Jena, Germany) for 18 s, at 9000 mJ/cm^2^. The plates were incubated at room temperature for 48 h until individual colonies could be observed. These colonies were individually picked into 96-well plates using sterile toothpicks. Mutants that failed to develop sclerotia were identified in approximately 7 days and were transferred to separate PDA plates for further phenotypic analyses and confirmation.

### 2.3. S. sclerotiorum Genomic DNA Extraction and NGS Analysis

DNA extraction and NGS data analysis of mutants were performed as described previously [4]. Young mycelia grown for 3–5 days were collected from heritable mutants for DNA extraction using the cetyltrimethylammonium bromide (CTAB) method [37]. The genomic DNA isolated from each mutant was then examined to ensure little DNA degradation on agarose gels before sending for next-generation sequencing (NGS) by Novogene Co, Ltd. Paired-end library preparation, DNA-seq, and read quality control of samples were completed by Novogene, using Illumina sequencer NovaSeq 6000, leading to 14 to 17 million clean reads per mutant sample. To identify the mutation sites, the derived NGS reads were first mapped to the reference genome of WT *S. sclerotiorum* 1980 strain by BWA-MEM algorithm [38]. The aligned mutant reads were then transformed into a binary format by SAMtools [39]. The resulting binary alignment map (BAM) files were analyzed by GATK 3.7 with its best practices for variant calling (SNPs + Indels) [40]. Mutations identified in repetitive sequences were manually removed from further analyses, as they are unlikely to be causal mutations.

### 2.4. Target Gene Knockout

A homologous recombination-based approach was employed to construct gene replacement cassettes for *SsATG1*, *SsATG9*, *SsATG2*, *SsATG5*, *SsATG4*, and *SsATG26* and subsequent generation of the KO mutants, following the protocol as described [4]. In short, the replacement cassettes consisting of the gene upstream sequence (1400–1600 base pairs [bp]), the hygromycin resistance gene (*HYG*) (1504 bp), and the gene downstream sequence were generated through PCR amplification of WT *S. sclerotiorum* genomic DNA or pCH-EF-1 containing the *HYG* sequence. Individual fragments were joined using double-joining PCR and the complete cassette was amplified further before transformation into WT *S. sclerotiorum* protoplasts. Positive transformants were screened and purified on PDA supplemented with 50 µg/mL hygromycin B. The KO mutants were deemed pure when PCR using 5F and 6R primer pairs could no longer amplify the WT band (400–600 bp). All primers used for PCR amplification are listed in Table S1.

### 2.5. Growth Rate Assessment and Colony Morphology Analysis of S. sclerotiorum

All WT and mutant strains of *S. sclerotiorum* were initially cultured on a PDA medium for 2–3 days. Mycelial agar disks were prepared using a sterile hole punch and transferred from the colony margin to the center of fresh PDA plates (92 × 16 mm, SARSTEDT, Nümbrecht, Germany). The radius of the mycelial colony was measured using a ruler every 12 h at room temperature until the mycelia reached the plate edges. Three independent colonies were measured for each strain. Colony morphology was documented 14 days post-inoculation to observe sclerotia formation.

### 2.6. Plant Infection Assay

Detached leaves of *Nicotiana benthamiana* were inoculated with fresh mycelial plugs of *S. sclerotiorum* (2 mm or 5 mm in diameter) from 2-day-old PDA plates. The leaves were placed on moistened paper towels inside trays covered with transparent lids and incubated in a growth chamber under controlled conditions (23 °C; 16 h light/8 h dark photoperiod). The ImageJ 1.46r (https://imagej.net/ij/, accessed on 1 December 2024)) area measuring tool with 1 cm calibration was used to measure and quantify lesion areas of leaves. To reduce error caused by natural leaf variation, normalized lesion sizes were calculated by dividing the mutant lesion area by the lesion area from the adjacent WT *S. sclerotiorum* inoculum. This way, mutant lesion areas were reported as a proportion of the adjacent WT inoculum. Three independent experiments, each with 5 replicates, were carried out with similar results.

### 2.7. Observation of Compound Appressoria Formation

Mycelial plugs (5 mm in diameter) collected from the edges of colonies were transferred onto glass slides placed on moistened paper towels and incubated at room temperature for 2 days. The development of compound appressoria was examined and recorded using a ZEISS light microscope (Zeiss, Jena, Germany).

### 2.8. S. sclerotiorum Hyphal Allelism Test

Fresh mycelial plugs of two alleles of the KO mutant and the UV mutant with the corresponding candidate gene mutation were placed on opposite sides on a new PDA plate. Sclerotia formation was recorded after 14 days. Allelism tests with two different UV mutants served as positive controls.

### 2.9. Generation of Ssatg5-1::GFP-ATG8 Fusion Line

To generate a homozygous *Ssatg5-1* KO mutant carrying GFP-ATG8, we used mycelial fusion followed by isolating single colonies from ascospores generated from apothecia. Such genetic manipulation is possible as the ascospores are haploid, although each ascospore contains two nuclei [41]. *SsATG8* N-terminally tagged with GFP was kindly provided to us by Dr. A. Wang [33]. Fresh mycelia (less than 7 days) of *Ssatg5-1* and WT *S. sclerotiorum* expressing GFP-ATG8 grown on PDA were mixed together and then transferred to glass containers containing fresh, sterile carrots. After 21 days, sclerotia were harvested from the carrot media, washed using water, and dried for 24 h. To generate apothecia, the sclerotia were sterilized for 2 min using 10% bleach, rinsed twice with water, and then placed into sterilized jars with gravel at 4 °C for 21 days. Afterwards, the sclerotia were incubated at 23 °C with a 16 h light/8 h dark photoperiod until apothecia became obvious. Ascospores from apothecia were collected upon cap maturity and they were spread onto PDA plates at 100 ascospores/mL. Individual colonies were picked into 96-well plates and colonies that phenocopied *Ssatg5-1* were observed under the fluorescence microscope to confirm the GFP-ATG8 signal. The obtained *Ssatg5-1*::GFP-ATG8 strain was used for further microscopic analysis.

### 2.10. Fluorescence Microscopy

Young mycelia, mycelial aggregates, pre-melanized sclerotia, and fully melanized sclerotia in WT background expressing GFP-ATG8 were imaged using a ZEISS light microscope (ZEISS AXIO Imager M2) equipped with X-cite series 120Q for fluorescence illumination (Excelitas Technologies, Waltham, MA, USA). These autophagosome structures could be observed during *S. sclerotiorum* growth on PDA between days 6 and 10. Melanized sclerotia were thinly sliced using a razor blade and squished using a microscope coverslip before imaging. *Ssatg5-1* expressing GFP-ATG8 was imaged at the young mycelia and mycelial aggregate stages. Images were taken under a 20× or 100× magnification with the scale bars shown at the bottom of each image.

### 2.11. Fluorescence Intensity Quantification

The mean fluorescence pixel value in single hyphae from young mycelia, mycelial aggregates, pre-melanized sclerotia, and melanized sclerotia expressing GFP-ATG8 was measured with ImageJ using the measurement tool. Background correction was applied by subtracting the mean background fluorescence pixel value from the single hyphae mean fluorescence pixel value. Five hyphae from 3 individual samples were analyzed with similar results.

### 2.12. Autophagosome Number Quantification

Autophagosomes in single hyphae from young mycelia, mycelial aggregates, premelanized sclerotia, and melanized sclerotia expressing GFP-ATG8 were identified as green puncta expressing a significant GFP-ATG8 signal and being less than 1 µm. The area of single hyphae was measured in ImageJ using the measurement tool and was used to calculate the number of autophagosomes/µm^2^. Five hyphae from 3 individual samples were analyzed with similar results.

### 2.13. Statistical Analysis

Statistical analysis was carried out using one-way ANOVA to compare the means of more than two *S. sclerotiorum* strains, followed by Dunnett’s post hoc test or Tukey’s post hoc test to determine which means were significant. To avoid the risk of making Type I errors, multiple Student’s t-tests to compare WT and the *Ssatg* mutants were not used. One-way ANOVA was used to compare the normalized lesion area, relative fluorescence intensity, and autophagosome number between the *Ssatg* mutants and WT. Dunnett’s post hoc test was used to determine which *Ssatg* mutant had significantly reduced lesion size compared to the WT control. Tukey’s post hoc test was used for comparing mean relative fluorescence intensities and autophagosome numbers to determine which mutants had significant differences from each other. Only the statistical significance between the normalized lesion area of WT and the *Ssatg* mutants was shown for quantifying the lesion areas. The WT lesion area was normalized to 1. Statistical significance was indicated using asterisks. *p*-values and sample numbers (*n*) are explained in the figure legends.

## 3. Results

### 3.1. S. sclerotiorum Mutants That Cannot Form Sclerotia Were Obtained from UV Mutagenesis

**Figure 1:**
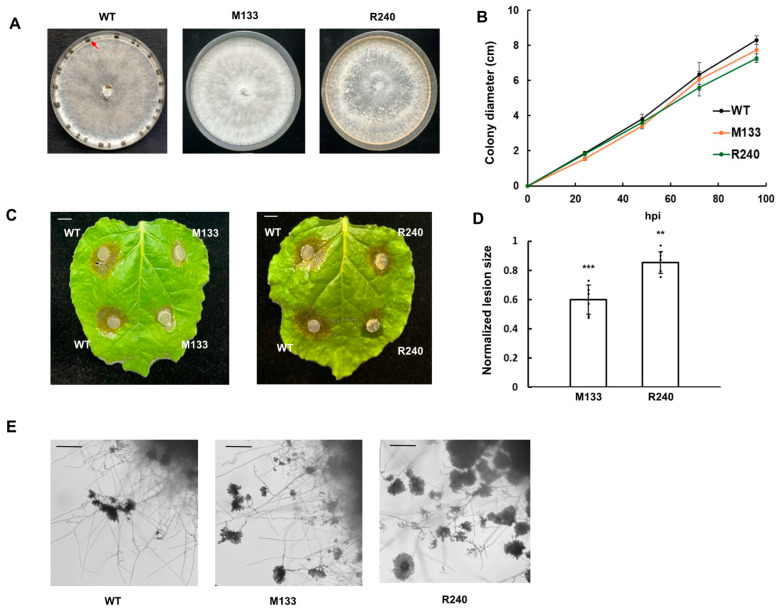
Phenotypic characterization of two UV mutants isolated in the WT 1980 background, M133 and R240. (**A**). Colony morphology of WT, M133 and R240 on PDA plates. The red arrow indicates sclerotia formation. The pictures were taken at 14 days post inoculation (dpi). (**B**). Mycelial growth of WT, M133, and R240 on PDA plates. The colony radius was measured every 24 h until the WT mycelia reached the plate edge. Three independent experiments were carried out with similar results. (**C**). Pathogenicity test of WT, M133, and R240 on detached *N. benthamiana* leaves. The picture was taken at 30 h post inoculation (hpi). Bar = 1 cm. (**D**). Quantification of the lesion size as in C caused by the indicated *S. sclerotiorum* mutant strains relative to the WT control at 30 hpi. Statistical significance compared to WT control is indicated by asterisks (** *p* < 0.01, *** *p* < 0.001). Three independent experiments were carried out with similar results. Error bars represent means ± SD (n = 6). (**E**). Compound appressoria formation of WT, M133, and R240. The mutants were transferred from PDA plates onto glass slides for compound appressoria observation. The pictures were taken at 2 dpi. Bar = 250 μm.

To identify genes involved in sclerotia formation in *S. sclerotiorum*, we performed a forward genetic screen with UV mutagenesis to systematically isolate and characterize mutants without sclerotia [4]. Two mutants, M133 and R240, displayed distinctive phenotypes, including clustered mycelial aggregates and an inability to produce sclerotia after 14 days (Figure 1A). Both M133 and R240 had similar vegetative growth rates as WT (Figure 1B).

To investigate potential changes in virulence, we inoculated M133 and R240 on WT *N. benthamiana* leaves and observed reduced necrotic lesion size compared to WT (Figure 1C,D). Despite these virulence defects, both mutants were able to form compound appressoria similar in morphology as WT (Figure 1E).

**Figure 2:**
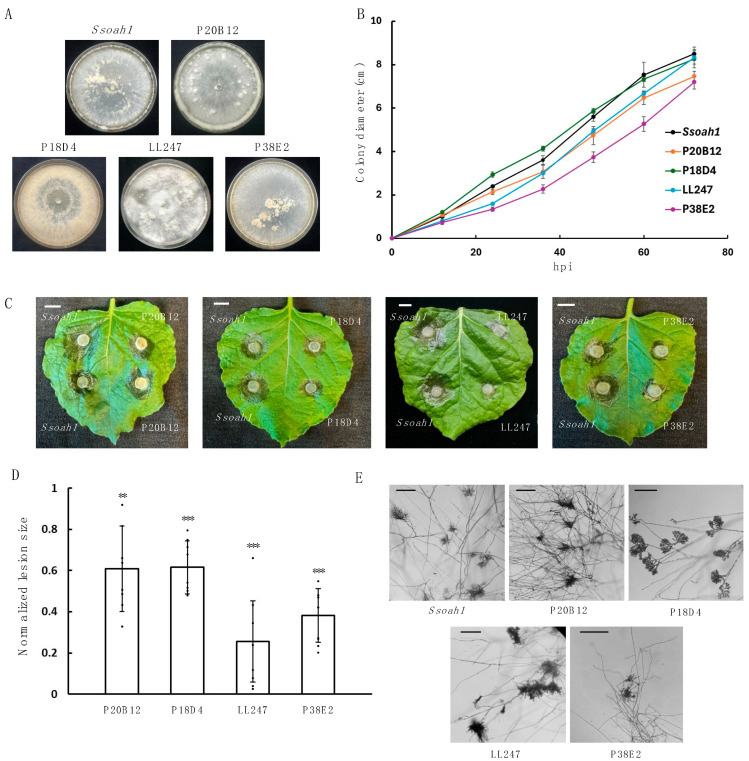
Phenotypic characterization of four *S. sclerotiorum* UV mutants isolated in the *Ssoah1* background—P20B12, P18D4, LL247, and P38E2. (**A**). Colony morphology of the oxalic acid biosynthesis CRISPR mutant *Ssoah1* and the UV mutants P20B12, P18D4, LL247, and P38E2 on PDA plates. Plate images were taken at 14 dpi. (**B**). Mycelial growth of the indicated *S. sclerotiorum* strains on PDA plates. The colony radius was measured every 12 h until the mycelia reached the plate edge. Three independent experiments were carried out with similar results. (**C**). Pathogenicity test of *Ssoa1h*, P20B12, P18D4, LL247, and P38E2 on detached *N. benthamiana* leaves. The picture was taken at 30 h post inoculation (hpi). Bar = 1 cm. (**D**). Quantification of the lesion size as in C caused by the indicated *S. sclerotiorum* mutant strains relative to the WT control at 30 hpi. Statistical significance compared to WT control is indicated by asterisks (** *p* < 0.01, *** *p* < 0.001). Three independent experiments were carried out with similar results. Error bars represent means ± SD (n = 8). (**E**). Compound appressoria formation of *Ssoah1*, P20B12, P18D4, LL247, and P38E2. The mutants were transferred from PDA plates onto glass slides for compound appressoria observation. The pictures were taken at 2 dpi. Bar = 250 μm.

Oxalic acid (OA) contributes to the virulence of *S. sclerotiorum*, as its absence renders the pathogen less virulent [42]. To identify OA-independent virulence factors in *S. sclerotiorum*, we additionally performed UV mutagenesis by using ascospores from the OA biosynthesis enzyme oxaloacetate acetylhydrolase (OAH) null mutant *Ssoah1* [34]. Mutants P20B12, P18D4, LL247, and P38E2 lost the ability to form sclerotia but exhibited similar vegetative growth rates as *Ssoah1* (Figure 2A,B). Markedly, all four mutants had significantly reduced virulence compared to *Ssoah1* despite maintaining appressoria formation (Figure 2C–E).

### 3.2. Distinct Autophagy-Related Genes Were Identified from Six UV Mutants

To identify the causal mutations for these mutants, genomic DNA of R240, M133, P20B12, P18D4, LL247, and P38E4 were extracted for NGS analysis, in accordance with an established method [4,35]. The analysis revealed the presence of *ATG* mutations in these mutants, as summarized in Table 1. As the *S. sclerotiorum Ssatg1* mutant, likely allelic to LL247, was previously shown to be required for sclerotia development and virulence [43], we hypothesized that other autophagy pathway components likely contribute to sclerotia formation. Therefore *SsATG4*, *SsATG9*, *SsATG1*, *SsATG2*, *SsATG5*, and *SsATG26* became the most likely candidates.

### 3.3. Knockouts of ATGs Exhibit Attenuated Sclerotia Formation and Virulence

**Figure 3:**
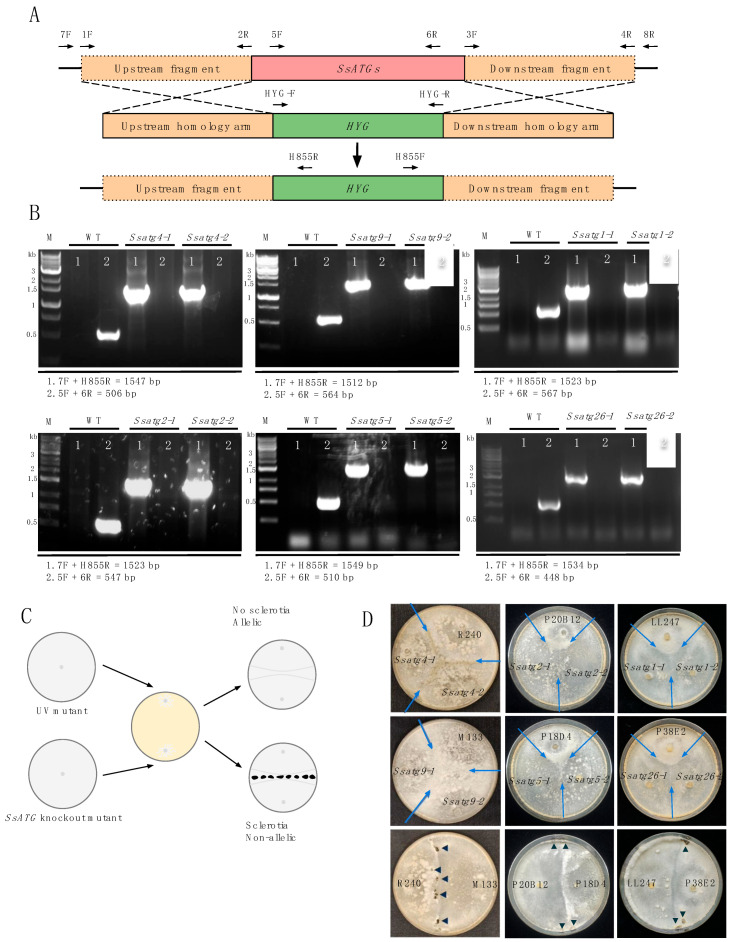
Generating *SsATG* deletion mutants as identified in Table 1. (**A**). Target gene KO strategy using homologous recombination. The *SsATG*s and *HYG* are shown as red and green rectangles, respectively. The primers specified with arrows were used to amplify the flanking regions or to genotype the KO transformants. (**B**). PCR verification of *SsATG4*, *SsATG9*, *SsATG2*, *SsATG5*, *SsATG1* and *SsATG26* gene KO. The genomic DNA extracted from WT *S. sclerotiorum* and two independent allelic KO mutants were used as PCR templates. Primer pair 1 was used to test the *HYG* gene insertion, and primer pair 2 was used to test the deletion of the *SsATG*s. Lane M is the DNA size ladder. (**C**). A diagram depicting the mutant pairing experiment designed for testing allelism of *S. sclerotiorum* mutants. (**D**). Hyphal alellism test of the UV mutant and two deletion KO alleles for *SsATG4*, *SsATG9*, *SsATG2*, *SsATG5*, *SsATG1* and *SsATG26*. Light blue arrows represent areas of overlapping mycelia between the mutants. Allelism tests between R240:M133, P20B12:P18D4, and LL247:P38E2 were used as positive controls. Dark blue arrows indicate sclerotia formation. The pictures were taken at 14 dpi.

To test whether the identified mutations in the ATGs are responsible for the phenotypic defects observed in UV mutants, we generated single-gene KO mutants for the six *SsATG*s (Table 1) in the WT background (Figure 3A). For each KO mutant, at least two independent deletion alleles were obtained. To verify successful gene KO, we performed PCR to ensure the purity of the deletions intended and the presence of the hygromycin-resistant gene in the target genes (Figure 3B).

In *S. sclerotiorum*, a hyphal fusion assay was previously established to determine whether different sclerotia-deficient mutants are allelic with each other [44]. This assay uses the ability of *S. sclerotiorum* to form stable heterokaryons after hyphal fusion [45]. In the case that fused strains are allelic, there should be no recovery of the WT phenotype. To confirm the allelic relationship between *Ssatg* KO mutants and their respective original UV mutant, we inoculated two alleles of the *Ssatg* KO and the respective UV mutant on a single PDA plate (Figure 3C). In the areas of fused mycelia, no sclerotia formation was observed between the UV mutant and its corresponding KO mutants, which indicated that they were allelic. In contrast, the fusion between different UV mutants, such as R240 and M133, displayed sclerotia formation, demonstrating that the individual UV mutants were not allelic and could complement each other (Figure 3D). These results confirmed that the developmental and virulence defects we observed in the UV mutants were indeed caused by the mutations in the *SsATG* genes and not due to off-target gene mutations.

**Figure 4:**
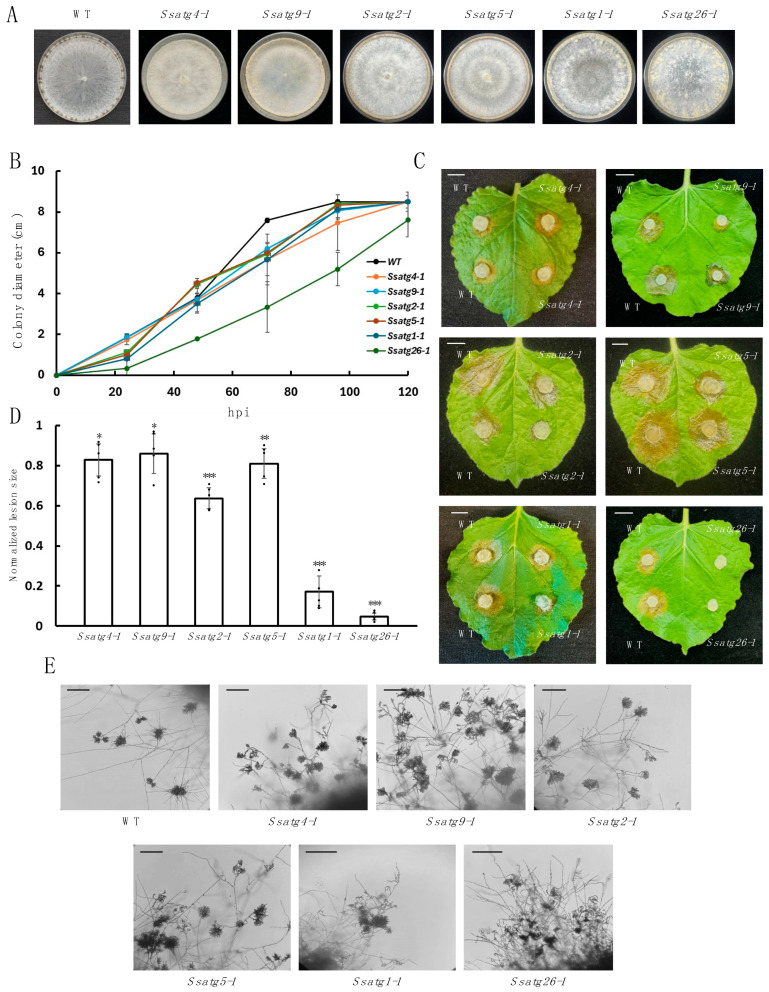
Phenotypic analysis of the *Ssatg* KO mutants. (**A**). Colony morphology of WT, *Ssatg4-1*, *Ssatg9- 1*, *Sssatg2-1*, *Ssatg5-1*, *Ssatg1-1*, and *Ssatg26-1* on PDA plates. Plate images were taken at 14 dpi. (**B**). Mycelial growth of the indicated *S. sclerotiorum* strains on PDA plates. The colony radius was measured every 24 h until the mycelia reached the plate edge. Three independent experiments were carried out with similar results. (**C**). Pathogenicity test of the indicated genotypes on detached *N. benthamiana* leaves. The picture was taken at 30 h post inoculation (hpi). Bar = 1 cm. (**D**). Quantification of the lesion size as in C caused by the indicated *S. sclerotiorum* genotypes relative to the WT control at 30 hpi. Statistical significance compared to WT control is indicated by asterisks (* *p* < 0.05, ** *p* < 0.01, *** *p* < 0.001). Three independent experiments were carried out with similar results. Error bars represent means ± SD (n = 5). (**E**). Compound appressoria formation of indicated genotypes. The mutants were transferred from PDA plates onto glass slides for compound appressoria observation. The pictures were taken at 2 dpi. Bar = 250 μm.

To better understand the function of *ATG*s in *S. sclerotiorum*, we examined the vegetative growth rate and inoculated the six *Ssatg* mutants on detached *N. benthamiana* leaves (Figure 4A–C). The virulence of the *Ssatg4*, *Ssatg9*, *Ssatg2*, and *Ssatg5* mutants were reduced compared to WT, but to a lesser degree than *Ssatg1* and *Ssatg26* (Figure 4C,D). Yet *Ssatg1* seemed to maintain the ability to penetrate the host, as shown by its appressoria formation (Figure 4C,D). Interestingly, the *Ssatg26* mutant grew more slowly and exhibited less virulence than the other *Ssatg* mutants, but it also displayed a similar compound appressoria phenotype as the WT (Figure 4A,E). As *Ssatg1* and *Ssatg26* had strongly reduced virulence despite maintaining appressoria formation, their infection process may be hindered post-penetration. To test this, *N. benthamiana* leaves were wounded before inoculation with *S. sclerotiorum*. This resulted in an increased lesion size of *Ssatg1* and *Ssatg26* (Figure S1). This suggests that the appressoria function of *Ssatg1* and *Ssatg26* mutants may have defects beyond its morphological similarity with the WT. Overall, these results suggest that *SsATG*s not only regulate sclerotia formation but also play a pivotal role in the infection process of *S. sclerotiorum*.

### 3.4. Autophagy Activity Is Dynamically Observed During Sclerotia Development

**Figure 5:**
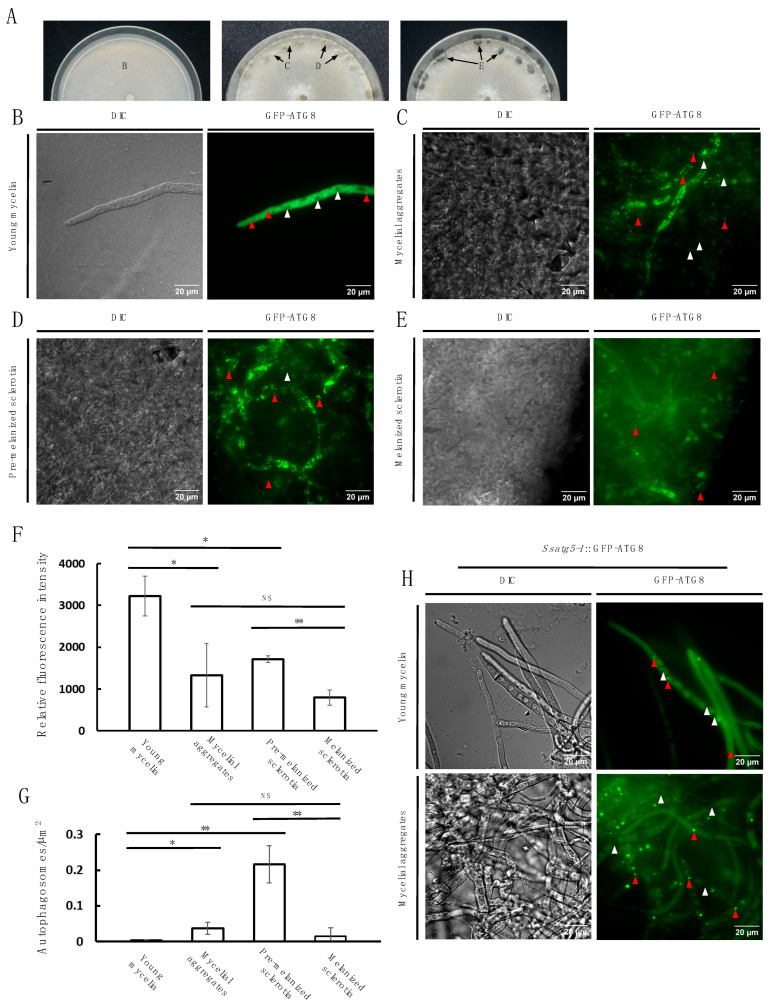
Microscopic analysis of *S. sclerotiorum* sclerotia development. (**A**). Time course of *S. sclerotiorum* vegetative growth and sclerotia development. Plate images of young mycelia (B), mycelial aggregates (**C**), pre-melanized sclerotia (**D**), and melanized sclerotia (**E**) were taken at 5, 6, and 7 dpi. (**B**). Brightfield (DIC) and fluorescence (GFP-ATG8) microscopy images of young mycelia. Red arrows indicate GFP-ATG8 concentrated in autophagosomes. White arrows indicate vacuoles. Bar = 20 μm. (**C**). Brightfield (DIC) and fluorescence (GFP-ATG8) microscopy images of mycelial aggregates. Red arrows indicate GFP-ATG8 concentrated in autophagosomes. White arrows indicate GFP-ATG8 deposition in vacuoles. Bar = 20 μm. (**D**). Brightfield (DIC) and fluorescence (GFP-ATG8) microscopy images of pre-melanized sclerotia. Red arrows indicate GFP-ATG8 concentrated in autophagosomes. White arrows indicate ATG8 deposition in vacuoles. Bar = 20 μm. (**E**). Brightfield (DIC) and fluorescence (GFP-ATG8) microscopy images of melanized sclerotia. Red arrows indicate GFP-ATG8 concentrated in autophagosomes. Bar = 20 μm. (**F**). Relative fluorescence intensity of WT young mycelia, mycelial aggregates, pre-melanized sclerotia, and melanized sclerotia expressing GFP-ATG8. Statistical significance between indicated groups is marked by asterisks. Three independent experiments were carried out with similar results. Error bars represent means ± SD (n = 5). (**G**). Autophagosome number per μm^2^ of hyphae in WT young mycelia, mycelial aggregates, pre-melanized sclerotia, and melanized sclerotia expressing GFP-ATG8. Statistical significance between indicated groups is marked by asterisks (* *p* < 0.05, ** *p* < 0.01). Three independent experiments were carried out with similar results. Error bars represent means ± SD (n = 5). (**H**). Brightfield (DIC) and fluorescence (GFP-ATG8) microscopy images of *Ssatg5-1*; GFP-ATG8 young mycelia and mycelial aggregates. Red arrows indicate GFP-ATG8 concentrated in autophagosomes. White arrows indicate vacuoles. Bar = 20 μm.

To confirm the involvement of autophagy in sclerotia formation, we used a previously generated GFP-ATG8 marker line to monitor macroautophagy [33] across four distinct developmental stages without any treatment: the young mycelial stage, the sclerotial initials stage with obvious mycelial aggregates, the pre-melanized sclerotia stage, and the melanized mature sclerotia stage (Figure 5A). During the young mycelial stage, GFP-ATG8 signal was dispersed in the cytoplasm with some GFP-ATG8 aggregated in autophagosomes (Figure 5B). During this stage, vacuoles were not observed with GFP-ATG8 signals (Figure 5B). In contrast, at the mycelial aggregate and pre-melanized sclerotia stages, less overall GFP-ATG8 signals were observed in the hyphae but there were increased autophagosome numbers, suggesting an increased autophagy activation (Figure 5C,D,F,G). In the fully melanized sclerotia, the GFP-ATG8 signal was observed in the cytoplasm and contained fewer autophagosomes, suggesting the termination of autophagy (Figure 5E,G). These microscopic observations support the contribution of autophagy during sclerotia formation. To further explore the possible defects in autophagy displayed in the KO mutants, we generated the *Ssatg5-1*::GFP-ATG8 line and observed the young mycelia and mycelial aggregate stages as these are stages with the most active autophagosome signals in WT. In *Ssatg5-1*, we observed GFP-ATG8 puncta in the young mycelia and puncta that seemed larger in the mycelial aggregates (Figure 5H). The larger puncta may be indicative of incomplete or non-functional autophagosomes [46]. Taken together, these results suggest that autophagy is actively involved in the sclerotia formation process and is dynamically regulated throughout the sclerotial developmental stages.

## 4. Discussion

**Figure 6:**
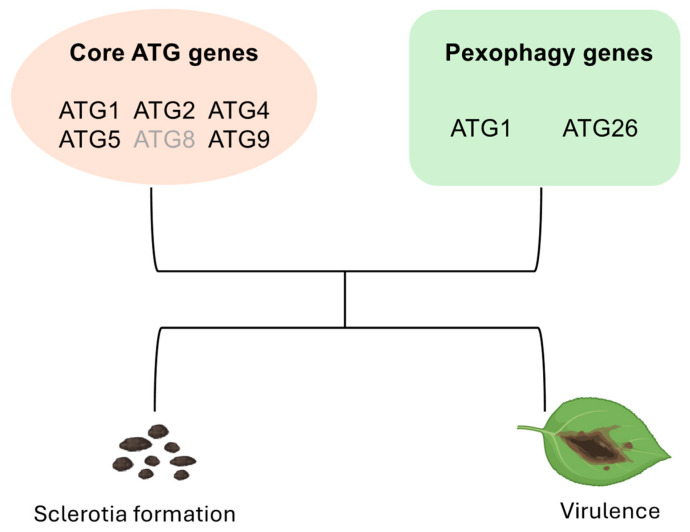
Proposed model of autophagy pathway components involved in *S. sclerotiorum* pathogenesis. In *S. sclerotiorum*, the core autophagy genes *ATG1*, *ATG2*, *ATG4*, *ATG5*, *ATG8*, and *ATG9* along with the pexophagy genes *ATG1* and *ATG26* are involved in sclerotia formation and virulence. Genes in grey were identified in a prior study, not in this work.

In eukaryotes, autophagy plays a crucial role in the nutrient recycling process under starvation, providing substances essential for survival [47]. Through autophagosomal nutrient recycling, nitrogen and carbon are released, enabling filamentous fungi to reproduce in nutrient-deficient environments [48,49,50]. In some fungi, sclerotia functioning as overwintering structures are often produced under nutrient-limiting conditions, allowing them to persist in harsh environments. In this study, six UV mutants that could not form sclerotia were identified through forward genetic analysis. By generating and characterizing individual *Ssatg* mutants, we confirmed that *ATG* genes in *S. sclerotiorum* play critical roles in sclerotia formation (Figure 6).

Using WT *S. sclerotiorum* expressing GFP-ATG8, we observed increased autophagosome numbers in the pre-melanized sclerotia compared to young mycelia and mycelial aggregates, suggesting increased autophagic activity during sclerotia formation (Figure 5F). Interestingly, there was decreased fluorescence intensity in the mycelial aggregates and the pre-melanized sclerotia compared to the young mycelia (Figure 5E). This may be explained by a lower overall fluorescence intensity in the hypha due to lipidated GFP-ATG8 localized to the autophagosomes, instead of being free in the cytoplasm. The GFP-ATG8 localization may exhibit reduced fluorescence excitation compared to its free state. Furthermore, there may be unequal rates of GFP-ATG8 lipidation and delipidation during sclerotia formation, which can alter the fluorescence intensity. In plants, *ATG5* is required for ATG8 recruitment to the phagophore and subsequent sealing of the autophagosome [25]. Interestingly, we could still observe autophagic structures in young mycelia and mycelial aggregates of *Ssatg5-1* expressing GFP-ATG8 (Figure 5H). In the closely related gray mold fungus *Botrytis cinerea*, silencing of *Bcatg5* led to the formation of incomplete autophagic structures that had disrupted lipid bilayers [46]. The GFP-ATG8 puncta in *Ssatg5-1* could be similar to these incomplete autophagosomes. Further careful cell biology and biochemical studies comparing GFP-ATG8 levels in autophagosomes versus lysosomal vacuoles will be required to quantify the autophagic process in WT and *Ssatg5*. Furthermore, TEM or higher resolution confocal microscopy is required to characterize the GFP-ATG8 puncta in *Ssatg5* and other mutant backgrounds. Collectively, these results suggest that autophagy, by degrading cellular components, seems to facilitate the redistribution of essential nutrients to developing sclerotia, thereby promoting their maturation and enhancing the survival of *S. sclerotiorum*. Without the autophagy genes, the organism cannot form sclerotia.

In addition to the TOR pathway, other signaling pathways also contribute to the onset of autophagy. In budding yeast, the Sty1 MAP kinase pathway has been shown to regulate the expression of autophagy-related genes under nutrient depletion conditions, and its deletion severely impairs the onset of autophagy [51]. In fission yeast, the *atg1* deletion mutant exhibits the same phenotype as the *pmk1* (PMK1 is the equivalent of STY1 in *Schizosaccharomyces pombe*) mutant, and overexpression of ATG1 was found to activate Pmk1 [52]. Furthermore, interactions between the TOR and cell integrity (CIP) pathways were observed [53,54], indicating a broader regulatory network linking autophagy and MAPK signaling. Recently, a MAPK signaling pathway equivalent to the yeast pheromone response pathway was characterized in *S. sclerotiorum*, consisting of *SsSte50*, *SsSte11*, *SsSte7*, and *Smk1* [55]. In the *ste50*, *ste11*, and *smk1* mutants, sclerotia formation was disrupted, resembling the phenotype observed in the *atg* mutants from our study (Figure 4A). Such similarity suggests that the autophagy and pheromone response pathways may regulate a common set of downstream genes essential for sclerotia formation in *S. sclerotiorum*. The relationship between the MAPK cascade and autophagy will be of interest in future research.

Appressoria are specialized infection structures used by fungi to penetrate host plant surfaces. Fungi such as *Magnaporthe oryzae* rely on substantial turgor pressure within appressoria, a process requiring functional *ATG* genes, to physically penetrate plant cuticles [56,57]. Compound appressoria are indispensable for the successful penetration in *S. sclerotiorum* [58]. In comparison, the *Ssatg2*, *Ssatg4*, *Ssatg5*, and *Ssatg9* mutants in our study exhibited no observable defects in compound appressoria formation (Figure 4E), consistent with the fact that they exhibited similar virulence on both wounded and unwounded leaves (Figure 4C and Figure S1). These ATGs likely contribute more to virulence post penetration. After cell wall penetration, autophagy likely contributes to the subsequent stages of infection, such as processing the nutrients acquired within host tissue, to promote virulence.

Pexophagy, a selective form of autophagy targeting peroxisomes for degradation, plays a crucial role in maintaining cellular homeostasis by regulating peroxisome numbers and removing damaged organelles [59]. In *Colletotrichum orbiculare*, deletion of *ATG26*, which encodes a sterol glucosyltransferase essential for peroxisome formation, results in severe defects in host invasion, despite normal appressorium development [60]. ATG1 also plays a role in pexophagy, and its absence impairs the delivery of peroxisomal cargoes into the vacuole via pexophagosomes [61]. In *S. sclerotiorum*, the *Ssatg1* and *Ssatg26* mutants exhibited pronounced defects in virulence (Figure 4A–D), although inoculation on wounded tobacco leaves resulted in increased lesion development (Figure S1). One possible explanation is that these mutants may have defects that impair appressorial function beyond visible morphological normality, thereby reducing the penetration efficiency of *Ssatg1* and *Ssatg26*. Our infection assays further indicate the critical role of pexophagy in the virulence of this fungus. Interestingly, less virulence was observed in *Ssatg26* than in *Ssatg1*. A possible explanation is that *S. sclerotiorum* exports large amounts of cell-wall-degrading enzymes, OA, and effectors. These proteins move through classical ER–Golgi vesicles and accumulate in sterol-rich lipid rafts at the hyphal tip. Deletion of *SsATG26* disrupts the biosynthesis of sterol glucosides (SGs) apart from pexophagy, which are crucial for fine-tuning the fluidity of lipid rafts [62]. In the absence of SGs, vesicle docking and fusion are impaired, leading to reduced secretion of pathogenic proteins and smaller lesion formation. Based on this hypothesis, future elucidation of the role of *SsATG26* and sterol glucosides in secretion and pathogenicity could reveal new targets for disease control strategies. Inhibitors that block sterol glucosylation or disrupt lipid raft function may impair the secretion of virulence factors, thereby reducing fungal infection. Additionally, host-induced gene silencing (HIGS) of *SsATG26* or other autophagy-related genes could be developed as a strategy to enhance crop resistance against *S. sclerotiorum*.

In a previous study, the *atg1* mutant of *S. sclerotiorum* exhibited defects in cell wall integrity when treated with various cell wall inhibitors [43]. This finding may explain why the *Ssatg1* mutants generated in both their study and ours failed to produce sclerotia. Additionally, they observed an absence of autophagosome formation in the mycelia of the *Ssatg1* mutant, further underscoring the critical role of *ATG1* in macroautophagy. Another core autophagy gene, *ATG8*, was also investigated [33]. Similar to other *atg* mutants in our study, the *atg8* mutant failed to form sclerotia. Moreover, the formation of appressoria, a crucial structure for successful host penetration during infection, was disrupted in the *atg8* mutant, leading to a complete loss of virulence. This phenotype is more severe than that observed in our core *atg* mutants, highlighting the irreplaceable and indispensable role of *ATG8* in virulence. However, it remains unclear whether this loss of virulence in the *atg8* mutant is due solely to impaired autophagy or *ATG8* has additional functions in *S. sclerotiorum*.

## 5. Conclusions

Through forward genetic screening and gene knockout validation, we identified core autophagy genes (*SsATG1*, *SsATG2*, *SsATG4*, *SsATG5*, and *SsATG9*) and the pexophagy related gene *SsATG26* as essential components for sclerotial development in *S. sclerotiorum*. Dynamic activation of autophagy during sclerotial maturation underscores the critical role of autophagy-mediated nutrient recycling in the formation of sclerotia. Collectively, our findings advance the understanding of fungal autophagy in pathogenesis and offer potential targets for crop protection.

## Figures and Tables

**Table 1:** Candidate mutation list.

Mutant	Nucleotide Change	Amino Acid Change	Reference Gene	Predicted Function
M133	T407C T356C A574T	F136S L119S M192L	sscle_01g000110 sscle_01g000110 sscle_05g044500	Mitochondrial substrate/solute carrier Mitochondrial substrate/solute carrier SIT4 phosphatase-associated protein
	G299A	G100E	sscle_09g072660	Nucleic acid-binding protein
	**C376T**	**R126STOP**	**sscle_11g082070**	**Autophagy-related protein ATG9**
	G766A	E256K	sscle_13g092650	Histidine phosphatase
	G843A	M281I	sscle_13g092670	Serine/threonine protein kinase
R240	A360T G1037A	Q120H G346E	sscle_03g027360 sscle_05g040590	None Acyl-coenzyme A diphosphatase NUDT19
	C1120T	Q374STOP	sscle_09g070220	Fructose-2,6bisphosphatase
	**C485T**	**S162F**	**sscle_12g089570**	**Autophagy-related protein ATG4**
P20B12	A938G **G4417A**	K313R **A1473T**	sscle_02g020610 **sscle_03g023750**	RNA-dependent RNA polymerase **Autophagy-related protein ATG2**
	C167T	S56F	sscle_04g034370	Glycosyltransferase
	648delT	Y216STOP	sscle_08g062260	Sterol reductase
P18D4	A5309G	K1770R	sscle_07g055370	Class II myosin
	**T656G**	**L219STOP**	**sscle_08g066910**	**Autophagy-related protein ATG5**
LL247	A2776G	N92D6	sscle_04g032930	E3 ubiquitin-protein ligase
	**C1066T**	**R356STOP**	**sscle_12g087380**	**Autophagy-related protein ATG1**
P38E2	C940T T271G	P314S Y91D	sscle_01g004320 sscle_01g010390	Not known Mitochondrial chaperone BCS1
	T547G	L183V	sscle_09g069660	Not known
	**A3875G**	**Q1292R**	**sscle_11g081870**	**Autophagy-related protein ATG26**
	G515A	G172E	sscle_11g086620	Nucleoporin

Genes highlighted in **bold** are candidate causal *atg* genes.

## Data Availability

All data are available in the main text or the Supplementary Materials. X.L. is responsible for distribution of materials integral to the findings presented in this paper.

## Supplementary Materials

The following supporting information can be downloaded at: https://www.mdpi.com/article/10.3390/jof11050391/s1, Figure S1: Infection of *S. sclerotiorum* on prewounded *N. benthamiana* leaves; Table S1: The list of primers used in this study.

## References

[1] Xia S., Xu Y., Hoy R., Zhang J., Qin L., Li X. (2019). The notorious soilborne pathogenic fungus Sclerotinia sclerotiorum: An update on genes studied with mutant analysis. Pathogens.

[2] Melzer M., Smith E., Boland G. (1997). Index of plant hosts of Sclerotinia minor. Can. J. Plant Pathol..

[3] Liang X., Rollins J.A. (2018). Mechanisms of broad host range necrotrophic pathogenesis in Sclerotinia sclerotiorum. Phytopathology.

[4] Xu Y., Ao K., Tian L., Qiu Y., Huang X., Liu X., Hoy R., Zhang Y., Rashid K.Y., Xia S. (2022). A forward genetic screen in Sclerotinia sclerotiorum revealed the transcriptional regulation of its sclerotial melanization pathway. Mol. Plant-Microbe Interact..

[5] Tariq V., Jeffries P. (1984). Appressorium formation by Sclerotinia sclerotiorum: Scanning electron microscopy. Trans. Br. Mycol. Soc..

[6] Yamamoto H., Zhang S., Mizushima N. (2023). Autophagy genes in biology and disease. Nat. Rev. Genet..

[7] Hollenstein D.M., Kraft C. (2020). Autophagosomes are formed at a distinct cellular structure. Curr. Opin. Cell Biol..

[8] Marzella L., Ahlberg J., Glaumann H. (1981). Autophagy, heterophagy, microautophagy and crinophagy as the means for intracellular degradation. Virchows Archiv. B Cell Pathol. Incl. Mol. Pathol..

[9] Ohsumi Y. (2014). Historical landmarks of autophagy research. Cell Res..

[10] Lynch-Day M.A., Klionsky D.J. (2010). The Cvt pathway as a model for selective autophagy. FEBS Lett..

[11] Oku M., Sakai Y. (2010). Peroxisomes as dynamic organelles: Autophagic degradation. FEBS J..

[12] Shintani T., Huang W.P., Stromhaug P.E., Klionsky D.J. (2002). Mechanism of cargo selection in the cytoplasm to vacuole targeting pathway. Dev. Cell.

[13] van Zutphen T., Veenhuis M., van der Klei I.J. (2008). Pex14 is the sole component of the peroxisomal translocon that is required for pexophagy. Autophagy.

[14] Noda N.N., Inagaki F. (2015). Mechanisms of autophagy. Annu. Rev. Biophys..

[15] Stjepanovic G., Davies C.W., Stanley R.E., Ragusa M.J., Kim D.J., Hurley J.H. (2014). Assembly and dynamics of the autophagy-initiating Atg1 complex. Proc. Natl. Acad. Sci. USA.

[16] Matsuura A., Tsukada M., Wada Y., Ohsumi Y. (1997). Apg1p, a novel protein kinase required for the autophagic process in Saccharomyces cerevisiae. Gene.

[17] Kabeya Y., Noda N.N., Fujioka Y., Suzuki K., Inagaki F., Ohsumi Y. (2009). Characterization of the ATG17–ATG29–ATG31 com-plex specifically required for starvation-induced autophagy in Saccharomyces cerevisiae. Biochem. Biophys. Res. Commun..

[18] Suzuki S.W., Yamamoto H., Oikawa Y., Kondo-Kakuta C., Kimura Y., Hirano H., Ohsumi Y. (2015). Atg13 HORMA domain recruits Atg9 vesicles during autophagosome formation. Proc. Natl. Acad. Sci. USA.

[19] Sekito T., Kawamata T., Ichikawa R., Suzuki K., Ohsumi Y. (2009). Atg17 recruits Atg9 to organize the pre-autophagosomal structure. Genes Cells.

[20] Mailler E., Guardia C.M., Bai X., Jarnik M., Williamson C.D., Li Y., Maio N., Golden A., Bonifacino J.S. (2021). The autophagy protein ATG9A enables lipid mobilization from lipid droplets. Nat. Commun..

[21] Baskaran S., Ragusa M.J., Boura E., Hurley J.H. (2012). Two-site recognition of phosphatidylinositol 3-phosphate by PROPPINs in autophagy. Mol. Cell.

[22] Osawa T., Noda N.N. (2019). ATG2: A novel phospholipid transfer protein that mediates de novo autophagosome biogenesis. Protein Sci..

[23] Rieter E., Vinke F., Bakula D., Cebollero E., Ungermann C., Proikas-Cezanne T., Reggiori F. (2013). Atg18 function in autophagy is regulated by specific sites within its β-propeller. J. Cell Sci..

[24] Kotani T., Kirisako H., Koizumi M., Ohsumi Y., Nakatogawa H. (2018). The Atg2-Atg18 complex tethers pre-autophagosomal membranes to the endoplasmic reticulum for autophagosome formation. Proc. Natl. Acad. Sci. USA.

[25] Le Bars R., Marion J., Le Borgne R., Satiat-Jeunemaitre B., Bianchi M.W. (2014). ATG5 defines a phagophore domain connected to the endoplasmic reticulum during autophagosome formation in plants. Nat. Commun..

[26] Hanada T., Noda N.N., Satomi Y., Ichimura Y., Fujioka Y., Takao T., Inagaki F., Ohsumi Y. (2007). The Atg12-Atg5 conjugate has a novel E3-like activity for protein lipidation in autophagy. J. Biol. Chem..

[27] Maruyama T., Noda N.N. (2018). Autophagy-regulating protease Atg4: Structure, function, regulation and inhibition. J. Antibiot..

[28] Martens S., Fracchiolla D. (2020). Activation and targeting of ATG8 protein lipidation. Cell Discov..

[29] Xie Z., Nair U., Klionsky D.J. (2008). ATG8 controls phagophore expansion during autophagosome formation. Mol. Biol. Cell.

[30] Yin Z., Chen C., Yang J., Feng W., Liu X., Zuo R., Wang J., Yang L., Zhong K., Gao C. (2019). Histone acetyltransferase MoHat1 acetylates autophagy-related proteins MoATG3 and MoATG9 to orchestrate functional appressorium formation and pathogenicity in *Magnaporthe oryzae*. Autophagy.

[31] Liu X.H., Zhao Y.H., Zhu X.M., Zeng X.Q., Huang L.Y., Dong B., Su Z.Z., Wang Y., Lu J.P., Lin F.C. (2017). Autophagy-related protein MoATG14 is involved in differentiation, development and pathogenicity in the rice blast fungus *Magnaporthe oryzae*. Sci. Rep..

[32] Yang X., Zhang L., Xiang Y., Du L., Huang X., Liu Y. (2020). Comparative transcriptome analysis of Sclerotinia sclerotiorum revealed its response mechanisms to the biological control agent, Bacillus amyloliquefaciens. Sci. Rep..

[33] Zhang H., Li Y., Lai W., Huang K., Li Y., Wang Z., Chen X., Wang A. (2021). SsATG8 and SsNBR1 mediated-autophagy is required for fungal development, proteasomal stress response and virulence in Sclerotinia sclerotiorum. Fungal Genet. Biol..

[34] Liang X., Liberti D., Li M., Kim Y.T., Hutchens A., Wilson R., Rollins J.A. (2015). Oxaloacetate acetylhydrolase gene mutants of Sclerotinia sclerotiorum do not accumulate oxalic acid, but do produce limited lesions on host plants. Mol. Plant Pathol..

[35] Zhang C., Xu Y., Li L., Wu M., Fang Z., Tan J., Rollins J.A., Lin H., Huang X., Mansfield S.D. (2025). A GDP-mannose-1-phosphate guanylyltransferase as a potential HIGS target against Sclerotinia sclerotiorum. PLoS Pathog..

[36] Athukorala S.N., Fernando W.D., Rashid K.Y., de Kievit T. (2010). The role of volatile and non-volatile antibiotics produced by Pseudomonas chlororaphis strain PA23 in its root colonization and control of Sclerotinia sclerotiorum. Biocontrol. Sci. Technol..

[37] Cubero O., Crespo A., Fatehi J., Bridge P. (1999). DNA extraction and PCR amplification method suitable for fresh, herbarium-stored, lichenized, and other fungi. Plant Syst. Evol..

[38] Li H. (2013). Aligning sequence reads, clone sequences and assembly contigs with BWA-MEM. arXiv.

[39] Li H., Handsaker B., Wysoker A., Fennell T., Ruan J., Homer N., Marth G., Abecasis G., Durbin R., 1000 Genome Project Data Processing Subgroup (2009). The sequence alignment/map format and SAMtools. Bioinformatics.

[40] Van der Auwera G.A., Carneiro M.O., Hartl C., Poplin R., Del Angel G., Levy-Moonshine A., Jordan T., Shakir K., Roazen D., Thibault J. (2013). From FastQ data to high-confidence variant calls: The genome analysis toolkit best practices pipeline. Curr. Protoc. Bioinform..

[41] Li X., Tian L., Xu Y., Tan J., Li J., O’Neil N., Hirst M., Hieter P., Zhang Y. (2025). Distribution of haploid chromosomes into separate nuclei in two pathogenic fungi. Science.

[42] Liang X., Moomaw E.W., Rollins J.A. (2015). Fungal oxalate decarboxylase activity contributes to Sclerotinia sclerotiorum early infection by affecting both compound appressoria development and function. Mol. Plant Pathol..

[43] Jiao W., Yu H., Chen X., Xiao K., Jia D., Wang F., Zhang Y., Pan H. (2022). The SsATG1 activating autophagy is required for sclerotia formation and pathogenicity in Sclerotinia sclerotiorum. J. Fungi.

[44] Xu Y., Qiu Y., Zhang Y., Li X. (2023). A cAMP phosphodiesterase is essential for sclerotia formation and virulence in Sclerotinia sclerotiorum. Front. Plant Sci..

[45] Ford E., Miller R., Gray H., Sherwood J. (1995). Heterokaryon formation and vegetative compatibility in Sclerotinia sclerotiorum. Mycol. Res..

[46] Li Y., Shu P., Xiang L., Sheng J., Shen L. (2023). CRISPR/Cas9-Mediated SlATG5 mutagenesis reduces the resistance of tomato fruit to Botrytis cinerea. Foods.

[47] Onodera J., Ohsumi Y. (2005). Autophagy is required for maintenance of amino acid levels and protein synthesis under nitrogen starvation. J. Biol. Chem..

[48] Deng Y.Z., Ramos-Pamplona M., Naqvi N.I. (2009). Autophagy-assisted glycogen catabolism regulates asexual differentiation in Magnaporthe oryzae. Autophagy.

[49] Richie D.L., Askew D.S. (2008). Autophagy in the filamentous fungus Aspergillus fumigatus. Methods Enzymol..

[50] Liu N., Zhu M., Zhang Y., Wang Z., Li B., Ren W. (2022). Involvement of the autophagy protein Atg1 in development and virulence in Botryosphaeria dothidea. J. Fungi.

[51] Corral-Ramos C., Barrios R., Ayté J., Hidalgo E. (2022). TOR and MAP kinase pathways synergistically regulate autophagy in response to nutrient depletion in fission yeast. Autophagy.

[52] Takasaki T., Utsumi R., Shimada E., Bamba A., Hagihara K., Satoh R., Sugiura R. (2023). Atg1, a key regulator of autophagy, functions to promote MAPK activation and cell death upon calcium overload in fission yeast. Microb. Cell.

[53] Madrid M., Vázquez-Marín B., Franco A., Soto T., Vicente-Soler J., Gacto M., Cansado J. (2016). Multiple crosstalk between TOR and the cell integrity MAPK signaling pathway in fission yeast. Sci. Rep..

[54] Cansado J., Soto T., Franco A., Vicente-Soler J., Madrid M. (2021). The fission yeast cell integrity pathway: A functional hub for cell survival upon stress and beyond. J. Fungi.

[55] Tian L., Li J., Xu Y., Qiu Y., Zhang Y., Li X. (2024). A MAP kinase cascade broadly regulates the lifestyle of Sclerotinia sclerotiorum and can be targeted by HIGS for disease control. Plant J..

[56] Liu X.H., Lu J.P., Zhang L., Dong B., Min H., Lin F.C. (2007). Involvement of a Magnaporthe grisea Serine/Threonine kinase gene, Mg ATG1, in appressorium turgor and pathogenesis. Eukaryot. Cell.

[57] Tan J., Zhao H., Li J., Gong Y., Li X. (2023). The devastating rice blast airborne pathogen Magnaporthe oryzae—A review on genes studied with mutant analysis. Pathogens.

[58] Bolton M.D., Thomma B.P., Nelson B.D. (2006). *Sclerotinia sclerotiorum* (Lib.) de Bary: Biology and molecular traits of a cosmopolitan pathogen. Mol. Plant Pathol..

[59] Sakai Y., Oku M., van der Klei I.J., Kiel J.A. (2006). Pexophagy: Autophagic degradation of peroxisomes. Biochim. Biophys. Acta (BBA)-Mol. Cell Res..

[60] Asakura M., Ninomiya S., Sugimoto M., Oku M., Yamashita S.I., Okuno T., Sakai Y., Takano Y. (2009). Atg26-mediated pexophagy is required for host invasion by the plant pathogenic fungus Colletotrichum orbiculare. Plant Cell.

[61] Komduur J.A., Veenhuis M., Kiel J.A. (2003). The Hansenula polymorpha PDD7 gene is essential for macropexophagy and microautophagy. FEMS Yeast Res..

[62] Pereira de Sa N., Del Poeta M. (2022). Sterylglucosides in Fungi. J. Fungi.

