# Supplementary material for: Autophagy-Related Proteins (ATGs) Are Differentially Required for Development and Virulence of Sclerotinia sclerotiorum
Source: J Fungi (Basel). 2025 May 19;11(5):391. doi: 10.3390/jof11050391 (PMC12113128; doi:10.3390/jof11050391)
Supplement: Supplementary file 1 [file jof-11-00391-s001.zip › jof-3605320-supplementary.pdf]

Supplemental Table S1. The list of primers used in this study

Name	Sequence	Purpose
Hygro-F	CCGGGATCCTCTAGAGTCG	Amplify hygromycin resistance gene HYG
Hygro-R	GAGCTGACATCGACACCAAC	
H855-F	GTCGATGCGACGCAATCGT	Check the insertion of hygromycin resistance gene HYG
H855-R	GAACCATCTTGTCAAACGAC	
ATG4-1F	TGAAGTGTGAAGCCTGCGAT	Knock out *SsATG4* gene
ATG4-2R	cgactctagaggatcccggGAGAGGGCTTTCGGGTCTTG	
ATG4-3F	gttggtgtcgatgtcagctcGGTTAAGGCACCGAGGAACA	
ATG4-4R	GAACCAGTTCGAGGCGCATA	
ATG4-5F	ATGATAAGGTCCGGGCAGAG	
ATG4-6R	TAAAGAAGGCGGCTTACCCA	
ATG4-7F	TGGTTCTGCAGACTCCAAGG	
ATG4-8R	GAACCAGTTCGAGGCGCATA	
ATG9-1F	AACGGTGGTCCCAGTCAATG	Knock out *SsATG9* gene
ATG9-2R	cgactctagaggatcccggATTGCCGCTCGTTCCTCTAC	
ATG9-3F	gttggtgtcgatgtcagctcCCGATGGAGGACGAGAATGG	
ATG9-4R	CGGAAAGAGAAATGTCCTGCC	
ATG9-5F	AAAAGATCTACCGCGGCGAT	

ATG9-6R	GACAATGACCGGAGCACAGA	
ATG9-7F	GAGTCCAGGCCAGCATGTC	
ATG9-8R	TCCTAGTCCGGGAAAGTGAAT	
ATG2-1F	ATTCAACACGGCGATTTAGG	Knock out *SsATG2* gene
ATG2-2R	cgactctagaggatcccggATGCGATCGTAGATGGGAGT	
ATG2-3F	gttggtgtcgatgtcagctcTTGCGCAGTCTCAGTTGTTT	
ATG2-4R	CGCTACTCAATCCTCCGAAC	
ATG2-5F	GTACGGGTGCATGGGATTAC	
ATG2-6R	GTCCACCATCACTAGCAGCA	
ATG2-7F	TGGGGATGATGAGGATAGTG	
ATG2-8R	CTGTCTTGTCATCGGGTCCT	
ATG5-1F	GTCAGGCGGAATGTAAGCAT	Knock out *SsATG5* gene
ATG5-2R	cgactctagaggatcccggAGCGCCAAGTAAGAGACTCG	
ATG5-3F	gttggtgtcgatgtcagctcAGGGTGACCGAGTTTAGCAA	
ATG5-4R	TCTCATGCCCTCCTCATCTC	
ATG5-5F	CTCTTAGGCCGATTTCATGC	
ATG5-6R	CCAATACCGCTTTGAAAGGA	
ATG5-7F	GTCCCCTCTTGTCTCGTTTG	

ATG5-8R	CACCGGTGTGGAACGTATTG	
ATG1-1F	CCCAGTGAGTGACATCAACCT	Knock out *SsATG1* gene
ATG1-2R	cgactctagaggatcccggCGTCGACTGGCATAGGGTAT	
ATG1-3F	gttggtgtcgatgtcagctcTCCAGGGCATCCAAGTAATC	
ATG1-4R	CAATACTAAGCCCCGAACCA	
ATG1-5F	ACGAGCCGATCTCTGGTCTA	
ATG1-6R	CGGTCCCTTTGTCTTGGTAA	
ATG1-7F	CCCCTTTTGCCTCTTCACTT	
ATG1-8R	ACCCCCTAAACCAACCAATC	
ATG26-1F	GAGGCCAATAGGAGATCACG	Knock out *SsATG26* gene
ATG26-2R	cgactctagaggatcccggCCTAGAACTGAACCGCGAAA	
ATG26-3F	gttggtgtcgatgtcagctcGCGTGCAACAGAAACAAAGA	
ATG26-4R	GCCATGATGAACTGAGCAGA	
ATG26-5F	GTACGGGATTTCGGCAAGTA	
ATG26-6R	TGGGGACAAAAGTTCCTCTG	
ATG26-7F	GGATGTCACGAACCACGTCT	
ATG26-8R	CATGGTGAAAGCTCGGAAAT	
